# Evaluation of pigment composition and antioxidant properties in the flesh of seven colored pummelo cultivars^☆^

**DOI:** 10.1016/j.fochx.2025.102705

**Published:** 2025-06-26

**Authors:** Peian Zhang, Quan Zhao, Yang Song, Huanchun Jin, Danelle Seymour, Yingyao Liu, Jun Chen, Dan Hu, Dongfeng Liu

**Affiliations:** a Zhejiang Institute of Subtropical Crops, Wenzhou, China; b Wenzhou Agricultural Technology Extension and Service Center, Wenzhou, China; c Department of Botany and Plant Sciences, University of California, Riverside, USA

**Keywords:** Carotenoid, Flavonoid, Anthocyanin, Pummelo, Antioxidant, Β-Carotene, Flesh color

## Abstract

The color of pummelo fruit is an important trait, but specific pigments' contribution remains unclear. This study analyzed seven colored pummelo cultivars, including three red (Sanhongmiyou, Gusangyou, and Chuhongyou); three pale-red (Ruby pummelo, Guanximiyou, and Feihongyou) and one orange (Jinjumiyou). Carotenoid, flavonoid, and anthocyanin content and antioxidant capacity were analyzed for each cultivar. In the red and pale-red cultivars, narirutin and lycopene were the most abundant carotenoids and flavonoids, respectively, while β-carotene was the most abundant carotenoids in the orange cultivar. Anthocyanins were also detected in the three red cultivars, with the most abundant anthocyanin being cyanidin. Of the three red cultivars, Chuhongyou contained the highest levels of cyanidin (19.6 μg·g^−1^) compared to Sanhongmiyou (5.8 μg·g^−1^) and Gusangyou (2.8 μg·g^−1^. Polymethoxylated flavones, lycopene, and cyanidin-3-O-(6”-O-coumaryl)-galactoside were significantly correlated with antioxidant capacity. This study summarizes the pigment composition and antioxidant capacity in fruit flesh across a spectrum of pigmented pummelos.

## Highlights

• Narirutin and lycopene were the dominant pigments in red and pale-red pummelo cultivars.
• Anthocyanins were detected for the first time in red pummelo pulp, and together with lycopene, influences the pulp color.
• The Jinjumiyou cultivar exhibited high β-carotene and a unique pigment composition.
• PMFs and anthocyanins impact antioxidant capacity.

## 1 Introduction

Pummelo (*Citrus maxima* (L.) Osbeck), is a large-fruited member of the Rutaceae family and is native to Southwest China, Southeast Asia, and Indochina (G. A. Wu et al., 2018). China is the largest producer of pummelo, accounting for 53.9 % of the world's annual production, with production areas located mainly in Fujian, Zhejiang, and Guangxi (C. Liu et al., 2015; Yin et al., 2023). The vast natural distribution and long-term cultivation of this fruit have resulted in a diverse range of pummelo varieties, with flesh colors ranging from white and yellow to orange, pink, and deep red (crimson) (C. Jiang, Zhang, Lin, Chen, & Lu, 2019). Variation in fruit color is mainly due to differences in the composition and concentration of pigmented substances such as carotenoids and flavonoids in the flesh, which exhibit strong antioxidant properties and bioactivity (Nishad et al., 2018).

Carotenoids are primarily C40 tetraterpenes and have a diverse range of functions in plant growth and development. Approximately 115 types of carotenoids have been identified in citrus (C. Liu et al., 2015; Ma, Zhang, & Kato, 2023). Carotenoids contain conjugated double bonds which enable the molecules to act as chromophores, imparting colors ranging from colorless to yellow or red, depending on the number of these bonds (Ma et al., 2023). As a result, specific carotenoid molecules can be associated with unique colors, including the pink lycopene, orange β-carotene, and yellow β-cryptoxanthin and zeaxanthin (Ma et al., 2023). Citrus has a wide spectrum of carotenoids that can be divided into three groups: β-cryptoxanthin-, violaxanthin-, and lycopene-abundant varieties, according to the carotenoid composition in the juice sacs of the flesh (Ikoma, Matsumoto, & Kato, 2016). For example, orange-colored mandarins and sweet oranges accumulate large amounts of β-cryptoxanthin and violaxanthin, respectively (Lado et al., 2019; Rodrigo, Cilla, Barbera, & Zacarias, 2015). Pummelo and lemon only accumulate small amounts of lutein, and some special red-flesh citrus budsport cultivars, such as the grapefruit cultivar “Star Ruby”, navel orange “Cara Cara”, and sweet orange “Hong Anliu” accumulate lycopene (Zhu et al., 2020). However, there is limited information on the amount of carotenoids in the flesh of pigmented pummelo cultivars.

More than 80 flavonoids from citrus fruits have been identified and characterized. Flavonoids and derived from a C6 (A ring)-C3 (C ring)-C6 (B ring) flavone skeleton (Y. Liu, Qian, et al., 2022). The hydrogen in the skeleton is usually substituted with various groups, such as hydroxyl, methoxyl, and glycosyl groups, resulting in large structural diversity of flavonoids, which can be categorized into flavanones, flavones, flavonols, and anthocyanins (Zhao, Wang, Lian, Xiao, & Zheng, 2020). Most of these flavonoids are pale yellow to yellow in color, though anthocyanins are red to blue-violet depending on the degree of hydroxylation on the B ring (i.e., orange/red for pelargonidin, red for cyanidin, and violet/blue for delphinidin). Methylation by hydroxylation further deepens the color (i.e., magenta for peonidin, purple/black for petunidin, and brown/dark brown for malvidin). Although most of the studies related to anthocyanins in citrus have focused on blood oranges and the detection of anthocyanin components in the young fruit and skin of pummelo, no investigation into whether anthocyanins are also present in red-fleshed pummelos has been conducted (H. Liu, Liu, Wu, Zheng, & Zhang, 2021).

Carotenoids and flavonoids are important antioxidants that affect the color of citrus flesh. Citrus fruits are a rich source of *O*-methylated flavonoids, and polymethoxylated flavones (PMFs) exist almost exclusively in citrus fruits (Saini et al., 2022). These molecules exhibit a wide range of bioactivities. In contrast to other flavonoids with free hydroxyl groups, PMFs exhibit a high degree of biological membrane permeability and membrane transport ability (Z. Peng et al., 2021). Flavanone neohesperidoses, such as naringin, neohesperidin, and neoeriocitrin have similar biological activities. They consist of a flavanone with neohesperidose (rhamnosyl-a-1,2 glucose) and contribute to the primary bitterness of citrus fruits (Chen et al., 2021). Rutinosides, such as narirutin, hesperidin, and eriocitrin, contain a basic flavanone combined with a disaccharide residue of rutinose (ramnosyl-a-1,6 glucose) and are tasteless (S. Liu, Lou, et al., 2022). Carotenoids are well-known for their antioxidant properties in normal cellular environments. Due to their high antioxidant activities, these carotenoids not only protect citrus fruits from abiotic and biotic factors but also protect humans from a wide range of chronic diseases (Karn et al., 2021; Zou, Xi, Hu, Nie, & Zhou, 2016).

In this study, seven pigmented pummelo cultivars were investigated to identify and quantify the carotenoid, flavonoid, and anthocyanin contents in their flesh. Liquid Chromatography-Tandem Mass Spectrometry (LC-MS/MS) was used to quantify each pigment, correlate their abundance, and determine the antioxidant capacity of each cultivar. Our work provides a better understanding of the contribution of different pigment molecules to flesh coloration in pummelo and guidance for breeders interested in developing pummelo cultivars with specific pigmentation characteristics.

## 2 Materials and methods

### 2.1 Materials

**Fig. 1:**
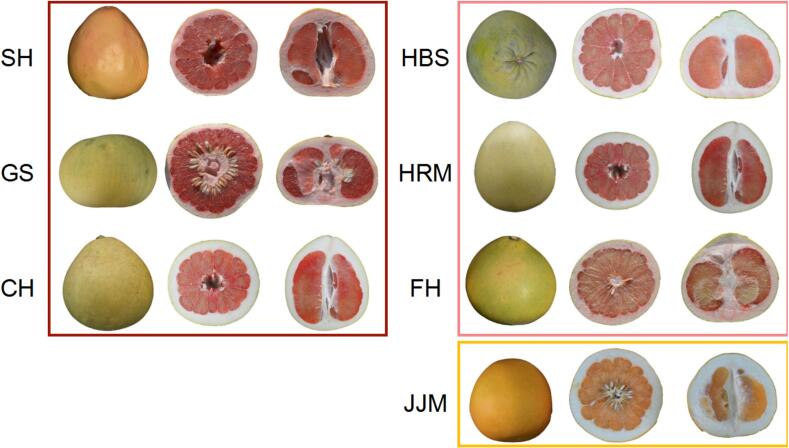
Pictorial view of the pummelo (*Citrus maxima*) fruits of seven cultivars with different pulp colors used in this study. Fruit samples were collected from pummelo germplasm resource nursery, Zhejiang Institute of Subtropical Crops, Wenzhou, China. Colored borders indicate red, light red and orange pummelo pulp color types. Cultivars name abbreviations: CH, Chuhongyou; FH, Feihongyou; GS, Gusangyou; HBS, Ruby pummelo; HRM, Red flesh Guanximiyou; JJM, Jinjumiyou; SH, Sanhongmiyou. (For interpretation of the references to color in this figure legend, the reader is referred to the web version of this article.)

Mature fruit samples from the seven different cultivars were collected in October 2023 from the pummelo germplasm resource nursery at the Zhejiang Institute of Subtropical Crops in Wenzhou, China. The seven cultivars were classified into three color types: the red cultivars Sanhongmiyou (SH), Gusangyou (GS), and Chuhongyou (CH); the pale-red cultivars Ruby pummelo (HBS), Red flesh Guanximiyou (HRM), and Feihongyou (FH); and the orange cultivar Jinjumiyou (JJM) (Fig. 1). Fruits were cut in the longitudinal plane, and juice sacs were collected as flesh samples 2 cm from the edge of the rind and flash-frozen in liquid nitrogen for subsequent analysis.

### 2.2 Measurement of flesh color parameters

The color of the flesh was determined using a colorimeter (C-300; Minolta, Osaka, Japan), and tested at four points uniformly selected on the longitudinal plane of the fruit 2 cm from the rind. The low brightness (L*), redness-greeness (a*), and yellowness-blueness (b*) values were obtained using the colorimeter, with L* representing brightness or lightness (0 = black, 100 = white), a* representing redness–greenness (−a* = greenness, +a* = redness) and b* representing yellowness–blueness (−b* = blueness, +b* = yellowness). These values were then used to calculate both hue angle degree [H = arctan (b*/a*)], where 0° = red–purple, 90° = yellow, 180° = bluish–green, and 270° = blue, and citrus color index (CCI), which was calculated as follows: CCI = 1000·a*/L*·b* (Habibi, Guillen, Serrano, & Valero, 2021).

### 2.3 Analysis of carotenoid, flavonoid, and anthocyanin composition

Detection of carotenoid, flavonoid, and anthocyanin contents was performed using MetWare (http://www.metware.cn/) based on the LC-MS/MS platform (QTRAP 6500; AB Sciex, Framingham, USA), with three biological replicates for each analysis. Each sample was freeze-dried, ground into a powder (30 Hz, 1.5 min), and stored at −80 °C.

#### 2.3.1 Carotenoids

50 mg powder was weighted and extracted with 0.5 mL mixed solution of n-hexane: acetone: ethanol (1:1:1, *v*/*v*/v). The extract was vortexed for 20 min at room temperature. The supernatants were collected after centrifuged at 12000 r·min^−1^ for 5 min at 4 °C. After two extractions, the supernatant was evaporated to dryness under nitrogen, and reconstituted in a solution of methanol: MTBE. The solution was passed through a 0.22 μm filter and then analyzed with a LC-APCI-MS/MS system. LC: column, YMC C30 (3 μm, 100 mm × 2.0 mm i.d); solvent system, methanol: acetonitrile (1:3, v/v) with 0.01 % BHT and 0.1 % formic acid (A), methyl tert-butyl ether with 0.01 % BHT (B); gradient program, started at 0 % B (0–3 min), increased to 70 % B (3–5 min), then increased to 95 % B (5–9 min), finally ramped back to 0 % B (10–11 min); flow rate, 0.8 mL·min^−1^; temperature, 28 °C; injection volume: 2 μL. MS analysis was performed using the API 6500 Q TRAP LC/MS/MS System, equipped with an APCI Turbo Ion-Spray interface, operating in a positive ion mode and controlled by Analyst 1.6.3 software (AB Sciex). The APCI source operation parameters were as follows: ion source, APCI+; source temperature 350 °C; curtain gas (CUR) was set at 25.0 psi.

#### 2.3.2 Flavonoids

20 mg powder was weighted and extracted with 0.5 mL 70 % methanol. 10 μL internal standard (4000 nmol·L^−1^) was added into the extract as internal standards (IS) for the quantication. The extract was sonicated for 30 min and centrifuged at 12000 r·min^−1^ for 5 min at 4 °C. The sample extracts were analyzed using an UPLC-ESI-MS/MS system. The analytical conditions were as follows, UPLC: column, Waters ACQUITY UPLC HSS T3 C18 (100 mm × 2.1 mm i.d., 1.8 μm); solvent system, water with 0.05 % formic acid (A), acetonitrile with 0.05 % formic acid (B); The gradient elution program was set as follows: 0–1 min, 10 %–20 % B; 1–9 min, 20 %–70 % B; 9–12.5 min, 70 %–95 % B,12.5–13.5 min, 95 % B; 13.5–13.6 min, 95 %–10 % B,13.6–15 min, 10 % B; The flow rate was set at 0.35 mL·min^−1^ and the temperature was set at 40 °C. MS analysis was performed using the API 6500 Q TRAP LC/MS/MS System. The ESI source operation parameters were as follows: ion source, ESI+/−; source temperature 550 °C; IS was 5500 V (Positive), −4500 V (Negative); CUR was set at 35 psi.

#### 2.3.3 Anthocyanins

50 mg powder was weighted and extracted with 0.5 mL methanol/water/hydrochloric acid (500:500:1, *V*/V/V). Then the extract was vortexed for 5 min and ultrasound for 5 min and centrifuged at 12000 r/min for 3 min at 4 °C. The residue was re-extracted by repeating the above steps again under the same conditions. The analytical conditions were as follows, UPLC: column, WatersACQUITY BEH C18 (100 mm × 2.1 mm i.d., 1.8 μm); solvent system, water (0.1 % formic acid): methanol (0.1 % formic acid); gradient program, 95:5 V/V at 0 min, 50:50 V/V at 6 min, 5:95 V/V at 12 min, hold for 2 min, 95:5 V/V at 14 min; hold for 2 min; flow rate, 0.35 mL·min^−1^; temperature, 40 °C. MS analysis was performed using the API 6500 Q TRAP LC/MS/MS System. The ESI source operation parameters were as follows: ion source, ESI+; source temperature 550 °C; IS was 5500 V; CUR was set at 35 psi.

### 2.4 Analysis of antioxidant activity

Hydroxyl radical scavenging activity (HRSA), 2,2′-diphenyl-1-picrylhydrazyl free radical scavenging capacity (DPPH), and ferric reducing antioxidant power (FRAP) were determined using the micro HRSA assay kit (BC1325, Solarbio, China), FRAP assay (BC1315, Solarbio, Beijing, China), and DPPH free radical scavenging ability assay kit (BC4750, Solarbio, Beijing, China), respectively, according to the manufacturers' guidelines.

### 2.5 Statistical analysis and data visualization

All experiments were conducted in at least triplicate biological replicates, and all data are presented as the means with standard deviation (SD), as calculated using Microsoft Excel (Microsoft Corporation, Albuquerque, NM, USA). Statistical analysis was conducted using one-way analysis of variance with SPSS software (version 19.0). Statistical significance was determined at *p* < 0.05. Duncan's test was used to analyze the correlations between seven colored pummelo cultivars. Orthogonal partial least squares discriminant analysis (OPLS-DA), principal component analysis (PCA) plots, circos relationship plots, heatmaps, and correlation analyses were performed using Metware Cloud, an online platform for data analysis (https://cloud.metware.cn).

## 3 Results

### 3.1 Color indicators of the flesh of pummelo cultivars

All color indicators of flesh color for the seven cultivars are summarized in Table 1. The red cultivars had relatively L* values and were significantly lower than those of the pale-red and orange cultivars. In contrast, the a* values were relatively high, with GS being the highest at 35.12 ± 3.67 and FH and JJM being relatively low at only 15.15 ± 4.55 and 19.24 ± 0.51, respectively. The b* value of the orange cultivar JJM was the highest among all cultivars at 30.85 ± 3.41, whereas the values for the other cultivars were relatively low. The H values were relatively high for FH and JJM, at 55.85 ± 6.05 and 57.86 ± 3.50, respectively, whereas the two red cultivars SH and GS were relatively low, at 31.91 ± 1.67 and 33.80 ± 1.25, respectively. The CCIs showed the same trend as the redness of the flesh, with GS having the highest CCI at 46.54 ± 2.38, followed by SH at 41.55 ± 1.87 and FH and JJM at 12.75 ± 3.02 and 11.79 ± 1.71, respectively.

**Table 1:** Color indicators of ripe pulp of seven different pummelo cultivars.

Cultivars	L*	a*	b*	H	CCI
SH	39.68 ± 2.23 e	32.07 ± 4.26 ab	22.00 ± 2.37 bc	31.91 ± 1.67 d	41.55 ± 1.87 b
GS	32.15 ± 1.03 f	35.12 ± 3.67 a	23.61 ± 3.65 b	33.80 ± 1.25 cd	46.54 ± 2.38 a
CH	43.34 ± 2.28 d	28.20 ± 2.88 bc	21.55 ± 3.23 bc	37.30 ± 2.52 c	30.45 ± 2.80 c
HBS	58.97 ± 0.90 ab	19.32 ± 2.34 de	20.21 ± 1.10 bc	46.39 ± 4.81 b	16.34 ± 2.83 e
HRM	47.55 ± 3.48 c	23.70 ± 1.58 cd	18.16 ± 0.40 c	37.51 ± 1.58 c	27.48 ± 1.20 d
FH	53.98 ± 2.34 b	15.15 ± 4.55 e	22.39 ± 5.58 bc	55.85 ± 6.05 a	12.75 ± 3.02 f
JJM	53.54 ± 1.57 b	19.24 ± 0.51 de	30.85 ± 3.41 a	57.86 ± 3.50 a	11.79 ± 1.71 f

Data are expressed as means ± standard deviation of triplicate samples; Different lowercase letters within a column represent significant differences between samples (*p* < 0.05). L*, low brightness; a*, redness-greeness; b*, yellowness-blueness; CCI, citrus color index; H, hue angle degree, SH, Sanhongmiyou; GS, Gusangyou; CH, Chuhongyou; HBS, Ruby Pummelo; HRM, Red flesh Guanximiyou; FH Feihongyou; JJM, Jinjumiyou.

### 3.2 Comparison of flavonoid and carotenoid components of pummelo cultivars

**Fig. 2:**
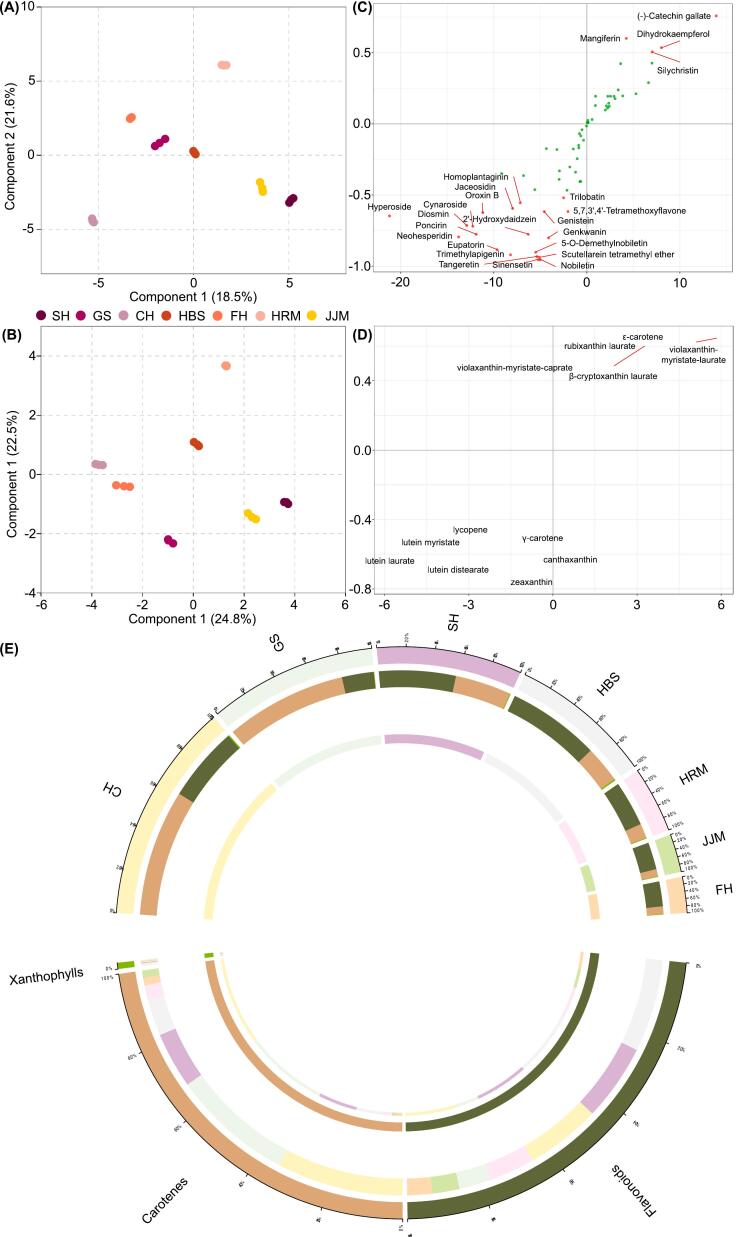
Flavonoid and carotenoid metabolites in the ripe pulp of seven pummelo cultivars. (A) and (B) OPLS-DA score plots for flavonoid and carotenoid metabolomics, respectively. (C) and (D) S-plot for flavonoid and carotenoid metabolomics, respectively. Red and green points indicate the metabolites with VIP values >1 and < 1, respectively. The VIP values of different metabolites are shown in Table S3 and S4. (E) Circos relationship plots for flavonoid and carotenoid metabolomics of seven pummelo cultivars. Abbreviations: CH, Chuhongyou; FH, Feihongyou; GS, Gusangyou; HBS, Ruby pummelo; HRM, Red flesh Guanximiyou; JJM, Jinjumiyou; SH, Sanhongmiyou; OPLS-DA, orthogonal partial least squares discriminant analysis; VIP, variable importance in projection. (For interpretation of the references to color in this figure legend, the reader is referred to the web version of this article.)

The flavonoid and carotenoid contents of the seven cultivars were detected by LC-MS/MS, and 69 and 27 metabolites were detected, respectively (Table S1, S2). Both the OPLS-DA score and PCA plots indicated that the flavonoid and carotenoid contents differed among the cultivars and illustrated distinct groupings of all seven cultivars (Fig. 2A, B, and Fig. S1A, B). Twenty-four and twelve markers were identified based on variable importance in projection (VIP) values >1 from the set of flavonoids and carotenoids, respectively. The seven metabolites with the highest VIP values among the flavonoids were sinensetin, nobiletin, scutellarein tetramethyl ether, tangeretin, trimethylapigenin, 5-*O*-demethylnobiletin, and eupatorin, all of which are PMFs and had VIP values >1.8 (Fig. 2C, Table S3). Among the carotenoids, zeaxanthin had a VIP value >1.7 and all other metabolites had a VIP value <1.5 (Fig. 2D, Table S4). Total flavonoid and carotenoid contents of the seven pummelo fruits were comparable, but the relative proportions of the two groups varied among the cultivars (Fig. 2E).

#### 3.2.1 Flavonoids

**Fig. 3:**
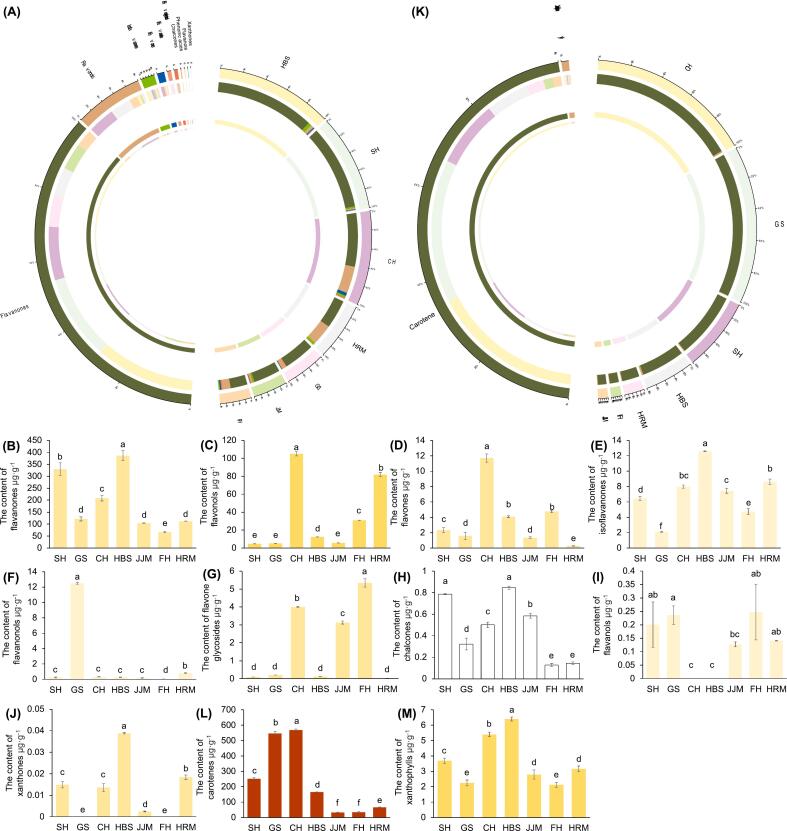
Classes of flavonoid and carotenoid metabolites found in the pulp of seven pummelo cultivars. Circos relationship plots for flavonoid (A) and carotenoid (K) metabolomics of seven pomelo cultivars, respectively. The content of flavanones (B), flavonols (C), flavones (D), isoflavanones (E), flavanonols (F), flavone glycosides (G), chalcones (H), flavanols (I), xanthones (J), carotenes (L), and xanthophylls (M), in the pulp of seven pummelo cultivars, respectively. The colors of the bar graph represent the approximate color type of such metabolites. Data are mean ± standard deviation (*n* = 3 biologically independent replicates). Different letters in the same row indicate significant differences according to Duncan's test (*p* < 0.05). Cultivars name abbreviations: CH, Chuhongyou; FH, Feihongyou; GS, Gusangyou; HBS, Ruby pummelo; HRM, Red flesh Guanximiyou; JJM, Jinjumiyou; SH, Sanhongmiyou. (For interpretation of the references to color in this figure legend, the reader is referred to the web version of this article.)

The highest flavonoid content was detected in the flesh of HBS, followed by SH, CH, HRM, GS, JJM, and FH (Fig. 3A). The flavanones were the most abundant flavonoid compound in each cultivar, with values ranging from 66.3 to 386.4 μg·g^−1^ (Fig. 3B), comprising more than 90 % of the flavonoids in HBS, SH, GS, and JJM. In contrast, in CH, HRM, and FH only 60 % of flavonoids were flavanones and than 30 % were flavonols (Fig. 3A–C). Flavonol content varied considerably among cultivars, with values ranging from 4.7 to 105.0 μg·g^−1^ (Fig. 3C). In addition, flavones, isoflavanones, flavanonols, and flavone glycosides were also detected in some cultivars, ranging from 0.28 to 11.7, 2.1 to 12.6, 0.01 to 12.5, and 0.02 to 5.3 μg·g^−1^, respectively (Fig. 3D–G). Chalcones, flavanols, and xanthones were relatively low in each cultivar, with the highest value for each being below 1 μg·g^−1^ (Fig. 3H–J).

**Fig. 4:**
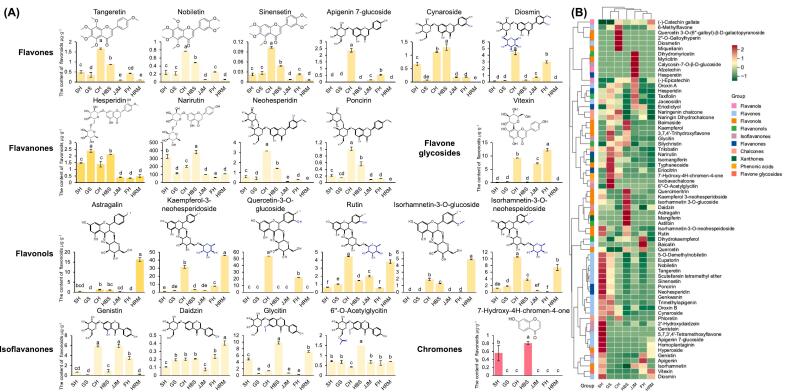
Analysis of flavonoid contents in seven pummelo cultivars. (A) Contents of major flavonoids. The colors of the bar graph represent the approximate color type of such flavonoid metabolites. Data are mean ± standard deviation (n = 3 biologically independent replicates). Different letters in the same row indicate significant differences according to Duncan's test (*p* < 0.05). (B) Hierarchical cluster analysis (HCA) of the flavonoid metabolomes. The columns and rows represent seven pummelo cultivars and 69 flavonoid metabolites, respectively. Each date was 3 biological replicates and normalised by row for *Z*-score. Cultivars name abbreviations: CH, Chuhongyou; FH, Feihongyou; GS, Gusangyou; HBS, Ruby pummelo; HRM, Red flesh Guanximiyou; JJM, Jinjumiyou; SH, Sanhongmiyou. (For interpretation of the references to color in this figure legend, the reader is referred to the web version of this article.)

Narirutin was both the most abundant flavonoid among the seven cultivars and the most abundant flavanone (Fig. 4A). Narirutin content was highest in HBS at 381.8 μg·g^−1^, followed by SH and CH at 327.7 and 203.0 μg·g^−1^, respectively. In contrast, FH had a relatively small amount narirutin at 65.7 μg·g^−1^. Other flavanones, such as hesperidin, neohesperidin, and poncirin, were found at relatively low levels. PMFs, including tangeretin, nobiletin, and sinensetin, were also detected. Notably, these PMFs showed a similar trend across cultivars (Fig. 4B). The highest levels of these PMFs were found in CH, followed by HBS, and relatively low levels were found in JJM and HRM. In addition, apigenin 7-glucoside and diosmin showed varied similarly across the seven cultivars, with relatively high levels in CH and FH. Flavonols were relatively and significantly higher in CH and HRM, with the highest content of kaempferol-3-neohesperidoside at 31.7 and 45.9 μg·g^−1^ in the two cultivars, respectively. The contents of the isoflavanones genistin and glycitin were relatively high but varied considerably in different cultivars (Fig. 4A). The genistin content was relatively high in CH, JJM, and FH at 5.9, 5.9, and 3.5 μg·g^−1^, respectively, whereas the glycitin content was relatively high in SH, HBS, and HRM at 4.9, 9.8, and 7.2 μg·g^−1^, respectively. Additionally, the isoflavanones in GS were present at very low levels, totaling only 0.36 μg·g^−1^. Furthermore, vitexin, a flavone glycoside, was detected only in CH, JJM and FH at 4.0, 3.1, and 5.3 μg·g^−1^, respectively, and 7-hydroxy-4 h-chromen-4-one, a chromone, was detected only in SH and HBS at 0.56 and 0.81 μg·g^−1^, respectively.

#### 3.2.2 Carotenoids

Carotenes and xanthophylls comprised 6 and 15 out of 21 total carotenoid molecules (Fig. 3K). The total amount of carotene was significantly higher than xanthophyll in all cultivars. The red cultivars SH, GS, and CH contained significantly higher carotene levels at 251.7, 546.3, and 569.0 μg·g^−1^, respectively, whereas the pale-red cultivars HBS and HRM had lower levels of carotene at 165.0 and 65.7 μg·g^−1^, respectively (Fig. 3L). The pale-red cultivar FH and orange cultivar JJM had the lowest levels of carotene at 34.3 and 32.3 μg·g^−1^, respectively, with no significant difference in the content between the two cultivars. The highest xanthophyll content was found in HBS at 6.4 μg·g^−1^, followed by CH, SH, JJM, HRM, GS, and FH (Fig. 3M).

**Fig. 5:**
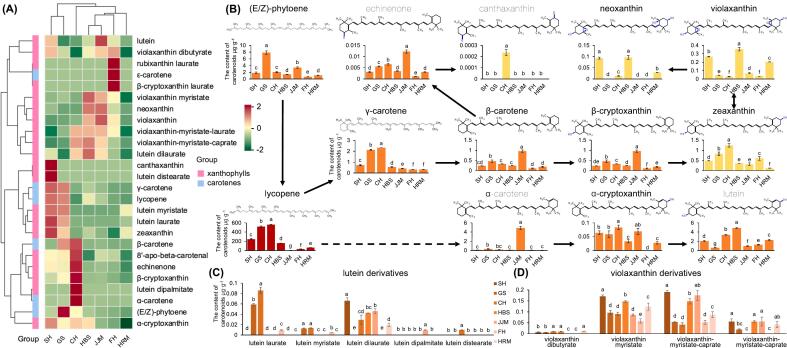
Carotenes and xanthophylls in seven pummelo cultivars. (A) Hierarchical cluster analysis (HCA) of carotenes and xanthophylls. The columns and rows represent seven pummelo cultivars and 27 carotene and xanthophyll metabolites, respectively. Each date was 3 biological replicates and normalised by row for Z-score. (B) Individual metabolite contents in the carotenoid biosynthesis pathway. The colors of the bar graph represent the approximate color type of such metabolites. (C) and (D) The contents of lutein and violaxanthin derivatives in the pulp of seven pummelo cultivars, respectively. Data are mean ± standard deviation (n = 3 biologically independent replicates). Different letters in the same row indicate significant differences according to Duncan's test (*p* < 0.05). Cultivars name abbreviations: CH, Chuhongyou; FH, Feihongyou; GS, Gusangyou; HBS, Ruby pummelo; HRM, Red flesh Guanximiyou; JJM, Jinjumiyou; SH, Sanhongmiyou. (For interpretation of the references to color in this figure legend, the reader is referred to the web version of this article.)

The most abundant carotene metabolite was lycopene, which exhibited a significantly higher level in the red cultivars SH, GS, and CH at 246.2, 519.2 and 561.3 μg·g^−1^, respectively, followed by the pale-red cultivars HBS, HRM and FH, at 159.7, 62.2 and 31.2 μg·g^−1^, respectively (Fig. 5). The orange cultivar JJM had the lowest lycopene content at 2.7 μg·g^−1^. Lycopene and its downstream metabolites γ-carotene and zeaxanthin showed similar trends across cultivars (Fig. 5A). β-carotene and β-cryptoxanthin, which are downstream of γ-carotene, as well as α-carotene, which has a similar molecular structure to that of γ-carotene, also showed similar trends across cultivars. The highest content of these metabolites was found in the orange cultivar JJM, which was significantly higher than that of other cultivars. β-carotene, β-cryptoxanthin, and α-carotene levels were 20.9, 0.96, and 4.9 μg·g^−1^, respectively. The contents of these metabolites were relatively high in the red cultivar GS, at 16.7, 0.46, and 0.34 μg·g^−1^, respectively (Fig. 5B).

#### 3.2.3 Correlation analysis of key metabolites in the flavonoid and carotenoid pathways

Chord diagrams of the correlations between the key flavonoid and carotenoid metabolites are shown in Fig. S2. Narirutin, the most abundant flavonoid, showed a significant positive correlation with naringin dihydrochalcone, trilobatin, 7-hydroxy-4 h-chromen-4-one, and violaxanthin myristate and a significant negative correlation only with quercetin. Lycopene, the most abundant carotenoid, was significantly positively correlated with four flavonoids, 2′-hydroxydaidzein, jaceosidin, oroxin B, and hesperidin, and one carotenoid, violaxanthin dibutyrate, and was significantly negatively correlated with lutein dipalmitate. β-carotene was significantly positively correlated with α-carotene, (E/Z)-phytoene, β-cryptoxanthin, and several orange carotenoids. Likewise, sinensetin, nobiletin, tangeretin, eupatorin, trimethylapigenin, scutellarein tetramethyl ether, and several other PMFs showed significant positive correlations.

### 3.3 Comparison of anthocyanin composition in the three red pummelo cultivars

To better understand whether the red color of red cultivars is associated with anthocyanins, the content of anthocyanins in flesh from SH, GS, and CH was examined. Significant differences were observed between all three red cultivars. Both OPLS-DA scores and PCA plots revealed variation in anthocyanin composition content among the three red cultivars. In total, 37 markers were identified based on VIP values >1 from the set of all identified anthocyanins (Fig. S3A—C). These included petunidin-3-O-glucoside, delphinidin-3-O-(coumaroyl)glucoside-5-O-galactoside, cyanidin-3-O-(6”-O-coumaryl)-galactoside, pelargonidin-3-O-(6-O-p-coumaroyl)-glucoside, and delphinidin-3-O-(coumaroyl)glucoside-5-O-glucoside, with VIP values of 1.30, 1.29, 1.22, 1.19, and 1.18, respectively, as well as several other abundant anthocyanins (Table S6).

**Fig. 6:**
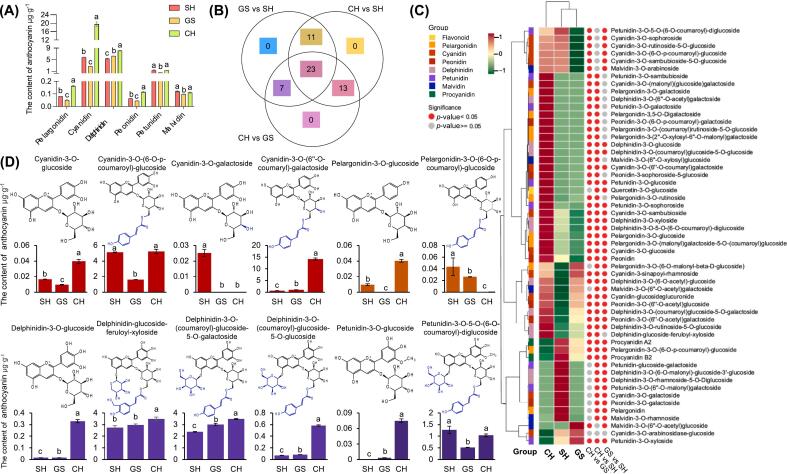
Anthocyanins in the pulp of three red pummelo cultivars. (A) Anthocyanin content by class. (B) Venn diagram of anthocyanins shared between the cultivars. (C) Hierarchal cluster analysis (HCA) of the anthocyanins. The columns and rows represent three red pummelo cultivars and 55 anthocyanins metabolites, respectively. Each date was 3 biological replicates and normalised by row for Z-score. The dots indicate the Pearson relationship of metabolites between the two cultivars, where red and gray dots indicate significant (*p* valus < 0.05) and non-significant (*p* valus ≥ 0.05) differences, respectively. (D) The contents of major anthocyanins. The colors of the bar graph represent the approximate color type of such metabolites. Data are mean ± standard deviation (n = 3 biologically independent replicates). Different letters in the same row indicate significant differences according to Duncan's test (*p* < 0.05). Cultivars name abbreviations: CH, Chuhongyou; GS, Gusangyou; SH, Sanhongmiyou. (For interpretation of the references to color in this figure legend, the reader is referred to the web version of this article.)

Fifty-four anthocyanin components were detected and classified into seven classes: cyanidin, delphinidin, malvidin, pelargonidin, peonidin, petunidin, and procyanidin, with 12, 11, 5, 10, 6, 8, and 2 metabolites in each class, respectively (Table S3). The anthocyanin content in CH was much higher than that in SH and GS, and each red cultivar was dominated by cyanidin and delphinidin. Notably, the proportion of the cyanidin and delphinidin was significantly different among the three cultivars, with cyanidin and delphinidin being the major anthocyanins in CH and GS, respectively, and the contents of these anthocyanins in SH were comparable (Fig. S3D). Cyanidin was the most abundant, at 5.8, 2.8, and 19.6 μg·g^−1^ in SH, GS, and CH, respectively, followed by delphinidin, at 5.3, 6.2, and 8.1 μg·g^−1^, respectively; the other types of anthocyanin were found in lower amounts (Fig. 6A). This correlation of anthocyanins was further underscored by high degree of overlap in anthocyanin molecules detected in each cultivar (Fig. 6B). Of the 48 anthocyanin metabolites evaluated, no unique metabolites were identified in any cultivar. Additionally, 23 metabolites were present across all three red cultivars, including cyanidin-3-O-glucoside, cyanidin-3-O-(6”-O-coumaryl)-galactoside, pelargonidin-3-O-glucoside, and petunidin-3-O-glucoside. The hierarchical cluster analysis revealed distinct patterns of abundance of anthocyanin metabolites across the three red cultivars (Fig. 6C). Anthocyanins in relatively high abundance included the red anthocyanins cyanidin-3-O-(6-O-p-coumaroyl)-glucoside and cyanidin-3-O-(6”-O-coumaroyl)-galactoside, the purple anthocyanins delphinidin-glucoside-feruloyl-xyloside and delphinidin-3-O-(coumaroyl)-glucoside-5-O-galactoside, and the blue-purple anthocyanins petunidin-3-O-5-O-(6-O-coumaroyl)-diglucoside (Fig. 6D). All five of these anthocyanins are coumaroylated at position 6 of their glycosides, while also retaining relatively small amounts of their most primitive glucosidated forms.

### 3.4 Correlation analysis of flavonoids and anthocyanins in red pummelos

**Fig. 7:**
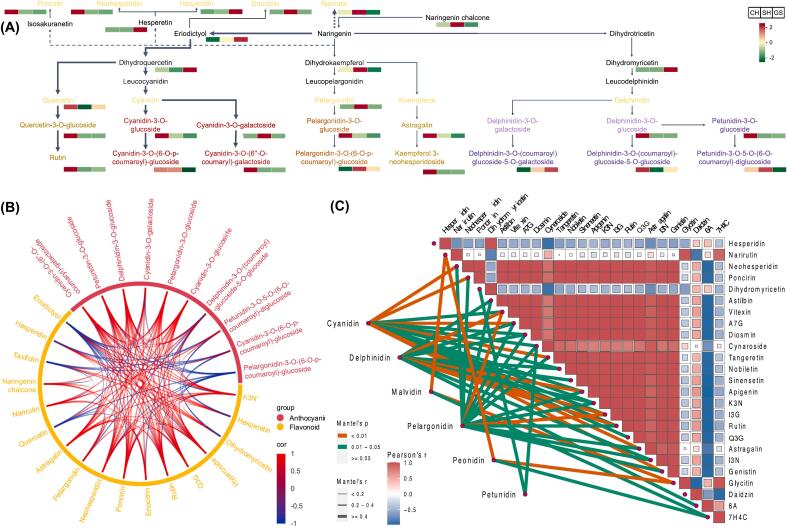
Analysis of flavonoids and anthocyanins in three red pummelo cultivars. (A) Individual metabolite contents in the flavonoid and anthocyanin biosynthesis pathway. The thickness of the arrow indicates the relative amount of metabolite content in the pathway. (B) The Pearson correlation of flavonoid and anthocyanin metabolites in related biosynthesis pathway. The color scale in the lower right corner shows Pearson correlation from −1 (red) to 1 (blue). (C) The square heatmap representing Pearson correlations for 25 major flavonoid metabolites, the color scale in the lower left corner shows Pearson correlation from −1 (red) to 1 (blue). The connecting lines on the lower left indicate the Mantel test results of different classes of anthocyanins with flavonoid metabolites, the thickness of the connecting lines indicates the coefficients of the overall correlation, and the color of the connecting lines indicates the results of the significance test of the correlation coefficients. Abbreviations: CH, Chuhongyou; GS, Gusangyou; SH, Sanhongmiyou; A7G, apigenin 7-glucoside; K3N, kaempferol 3-neohesperidoside; I3N, isorhamnetin-3-O-neohespeidoside; I3G, isorhamnetin 3-O-glucoside; 6 A, 6-*O*-acetylglycitin; 7H4C, 7-hydroxy-4H-chromen-4-one; Q3G, quercetin-3-O-glucoside. (For interpretation of the references to color in this figure legend, the reader is referred to the web version of this article.)

As indicated previously and illustrated in Fig. 7A, flavanone and anthocyanins biosynthesis, from naringin to eriodictyol, and downstream cyanidin and quercetin biosynthesis were the major flavonoid and anthocyanin biosynthesis pathways in the three red pummelo cultivars. In SH and GS, the biosynthesis of flavanone and anthocyanins was terminated at the eriodictyol to dihydromyricetin step, whereas CH was able to further synthesize large amounts of flavonoids and anthocyanins downstream of cyanidin and quercetin. Correlation analysis between 12 anthocyanins and 15 flavonoids in the above pathway revealed various associations. Cyanidin-3-O-(6”-O-coumaryl)-galactoside was significantly and positively correlated with astragalin, neohesperidin, poncirin, rutin, quercetin-3-O-glucoside, hyperoside, kaempferol-3-neohesperidoside, and many other anthocyanins were also significantly correlated with several flavonoids (Fig. 7B). Ultimately, a high degree of correlation was observed between anthocyanins and flavonoids. Furthermore, cyanidin-like anthocyanins, such as cyanidin-3-O-glucoside and cyanidin-3-O-galactoside, were also found to be significantly correlated with the above-mentioned flavonoids (*p* < 0.05), while delphinidin-like anthocyanins were significantly correlated with vitexin, apigenin, isorhamnetin-3-O-neohespeidoside, and rutin (*p* < 0.01). Narirutin was significantly correlated with malvidin and peonidin-like anthocyanins (*p* < 0.01) (Fig. 7C). In addition, PMFs were significantly correlated not only with cyanidin and delphinidin-like anthocyanins but also with pelargonidin-like anthocyanins (*p* < 0.05), though only tangeretin showed a significant correlation (*p* < 0.01) with cyanidin-like anthocyanins.

### 3.5 Antioxidant capacity of the flesh of pummelo cultivars

The antioxidant activities (HRSA, FRAP, and DPPH) of the flesh of the seven pummelo cultivars are presented in Table 2. The antioxidant activity of CH was significantly higher than that of other cultivars, at 89.53 ± 0.72 %, 226.28 ± 0.81 μmol·g^−1^, and 63.84 ± 0.29 μmol·g^−1^ for the HRSA, FRAP, and DPPH values, respectively. The GS and HBS also had relatively high HRSA values, at 68.67 ± 0.52 % and 62.02 ± 0.04 %, respectively, followed by SH and JJM at 50.57 ± 0.46 % and 50.26 ± 0.80 %, respectively, and HRM and FH at 31.92 ± 0.41 % and 15.74 ± 0.23 %, respectively. The FRAP and DPPH trends were similar across different cultivars. HBS exhibited the second highest antioxidant capacity only to CH, with FRAP and DPPH values of 182.84 ± 0.89 and 47.53 ± 0.77 μmol·g^−1^, respectively. Following HBS, the two other red cultivars SH and GS, showed FRAP values of 139.91 ± 0.39 and 140.01 ± 0.23 μmol·g^−1^, respectively, and DPPH values of 44.14 ± 0.21 and 39.71 ± 0.73 μmol·g^−1^, respectively. The two pale-red cultivars FH and HRM had relatively low FRAP values at 116.42 ± 0 and 116.78 ± 8.22 μmol·g^−1^, respectively, and DPPH values at 30.47 ± 0.06 and 22.06 ± 1.28 μmol·g^−1^, respectively. The orange cultivar JJM had the lowest FRAP and DPPH values at 52.01 ± 1.31 and 14.22 ± 0.01 μmol·g^−1^, respectively.

**Table 2:** The antioxidant activity in the pulp of seven pummelo cultivars.

Cultivars	HRSA/%	FRAP/μmol·g^−1^	DPPH/μmol·g^−1^
SH	50.57 ± 0.46 d	139.91 ± 0.39 c	44.14 ± 0.21 c
GS	68.67 ± 0.52 b	140.01 ± 0.23 c	39.71 ± 0.73 d
CH	89.53 ± 0.72 a	226.28 ± 0.81 a	63.84 ± 0.29 a
HBS	62.02 ± 0.04 c	182.84 ± 0.89 b	47.53 ± 0.77 b
JJM	50.26 ± 0.80 d	52.01 ± 1.31 e	14.22 ± 0.01 g
FH	15.74 ± 0.23 f	116.42 ± 0 d	30.47 ± 0.06 e
HRM	31.92 ± 0.41 e	116.78 ± 8.22 d	22.06 ± 1.28 f

Data are expressed as means ± standard deviation of triplicate samples; Different lowercase letters within a column represent significant differences between samples (*p* < 0.05). HRSA, Hydroxy free radical scavenging activity; FRAP, Ferric reducing antioxidant power; DPPH, 2,2′-diphenyl-1-picrylhydrazyl; SH, Sanhongmiyou; GS, Gusangyou; CH, Chuhongyou; HBS, Ruby Pummelo; HRM, Red flesh Guanximiyou; FH Feihongyou; JJM, Jinjumiyou.

### 3.6 Correlation of antioxidant capacity with pigmented components

The above results and those presented in Fig. S4 indicate that there is a correlation between color and antioxidant capacity. Since the pigmented components affecting flesh color may be correlated with antioxidant capacity, correlations between different carotenoid, flavonoid, and anthocyanin components and antioxidant capacities (with respect to HRSA, FRAP, and DPPH) were analyzed.

**Fig. 8:**
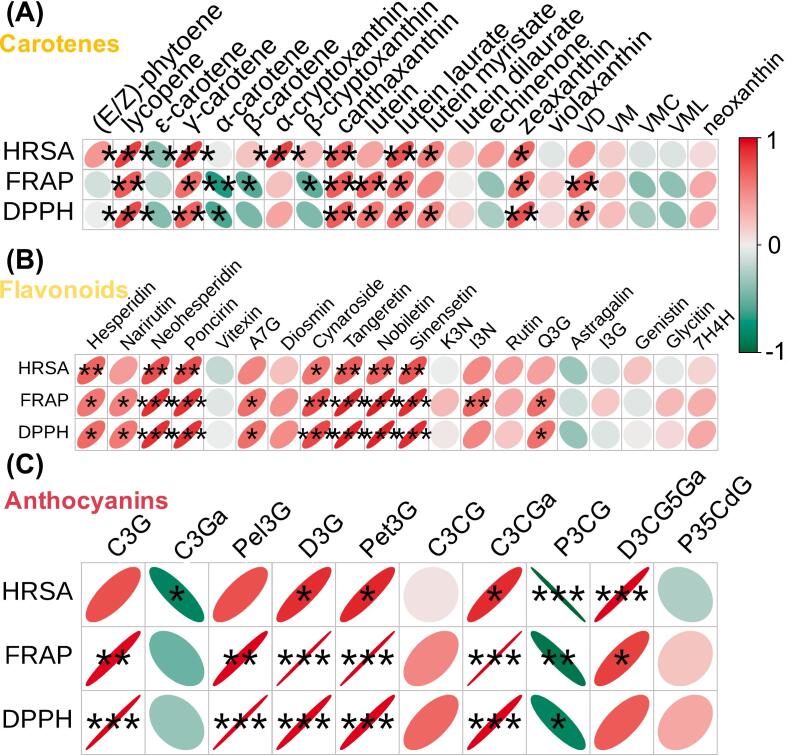
The Pearson correlation analysis of major pigments and antioxidant capacities (HRSA, FRAP and DPPH). (A) Correlation analysis with carotenes. (B) Correlation analysis with flavonoids. (C) Correlation analysis with anthocyanins. The color scale in the right corner shows Pearson correlation from −1 (green) to 1 (red). ‘*’, ‘**’, and ‘***’ indicate *p* values <0.05, 0.01, and 0.001, respectively. Abbreviations: HRSA, Hydroxy free radical scavenging activity; FRAP, Ferric reducing antioxidant power; DPPH, 2,2′-diphenyl-1-picrylhydrazyl free radical scavenging capacity; VD, violaxanthin dibutyrate; VM, violaxanthin myristate; VMC, violaxanthin-myristate-caprate; VML, violaxanthin-myristate-laurate; A7G, apigenin 7-glucoside; K3N, kaempferol 3-neohesperidoside; I3N, isorhamnetin-3-O-neohespeidoside; I3G, isorhamnetin 3-O-glucoside; 6 A, 6-*O*-acetylglycitin; 7H4C, 7-hydroxy-4H-chromen-4-one; Q3G, quercetin-3-O-glucoside; C3G, cyanidin-3-O-glucoside; C3Ga, cyanidin-3-O-galactoside; Pel3G, pelargonidin-3-O-glucoside; D3G, delphinidin-3-O-glucoside; Pet3G, petunidin-3-O-glucoside; C3CG, cyanidin-3-O-(6-O-p-coumaroyl)-glucoside; C3CGa, cyanidin-3-O-(6”-O-coumaryl)galactoside; P3CG, pelargonidin-3-O-(6-O-p-coumaroyl)-glucoside; D3CG5Ga, delphinidin-3-O-(coumaroyl)glucoside-5-O-galactoside; P35CdG, Petunidin-3-O-5-O-(6-O-coumaroyl)-diglucoside. (For interpretation of the references to color in this figure legend, the reader is referred to the web version of this article.)

Among the carotenoids, lycopene, γ-carotene, canthaxanthin, lutein laurate, and zeaxanthin were significantly and positively correlated with HRSA, FRAP, and DPPH (coefficient of determination *p* < 0.05; Fig. 8A). Lycopene had the highest correlation among all carotenoids (HRSA, *p* < 0.001; DPPH, *p* < 0.001; and FRAP, *p* < 0.01).

Among the flavonoids, three flavanones (hesperidin, neohesperidin, and poncirin) and four flavones (cynaroside, tangeretin, nobiletin, and sinensetin) were significantly and positively correlated with HRSA, FRAP, and DPPH (*p* < 0.05; Fig. 8B). Hesperidin, neohesperidin, poncirin, tangeretin, nobiletin, and sinensetin had *p* < 0.01 with HRSA, whereas except hesperidin had *p* < 0.01 with DPPH. In addition, narirutin, apigenin 7-glucoside, and quercetin-3-O-glucoside were significantly and positively correlated with FRAP and DPPH (*p* < 0.05).

Among the anthocyanins, delphinidin-3-O-glucoside, petunidin-3-O-glucoside, and cyanidin-3-O-(6”-O-coumaroyl)-galactoside were significantly and positively correlated with HRSA (*p* < 0.05), FRAP (*p* < 0.05), and DPPH (*p* < 0.05; Fig. 8C). However, pelargonidin-3-O-(6-O-p-coumaroyl)-glucoside showed a negative correlation with HRSA (*p* < 0.001), FRAP (*p* < 0.05), and DPPH (*p* < 0.01).

## 4 Discussion

With the increasing demand for the citrus fruit with a unique appearance and high quality, flesh color has become one of the most important target for improvement (Allan, 2019). As a result, an increasing number of pigmented pummelo cultivars have been identified and selected (Nishad et al., 2018); for example, red budsport cultivars including GS, CH, and HBS have become important for the Chinese pummelo consumer market (Tatmala, Ma, Zhang, Kato, & Kaewsuksaen, 2020). Previous studies have concluded that the pigmentation of red-fleshed citrus varieties, excluding blood oranges, is conferred mainly by lycopene (Butelli et al., 2017). The results of the present study also confirmed that the three red and three pale-red pummelo cultivars were rich in lycopene and lycopene content was positively correlated with the red color of the flesh (C. Jiang et al., 2019; Q. Jiang et al., 2022; Wang et al., 2019; Yang, Li, Kui, Sun, and Qiu, 2020). In SH, GS, and CH, the carotenoid content comprised 41 %, 78 %, and 60 %, of pigment-associated metabolites respectively, with more than 95 % of the carotenoids being lycopene. In the pale-red cultivars, although the carotenoid content was reduced to less than 30 % in all cultivars, lycopene still exceeded 90 % of total carotenoid content. In the orange-colored JJM, lycopene accounted for only 8 % of the carotenoids. However, whether flesh color is entirely determined by lycopene content remains in question, as a certain amount of anthocyanin has also been detected in red colored pummelos. Similarly to Tarocco blood orange, red pummelos were also abundant in cyanidin-like anthocyanin (Morales, Bermejo, Navarro, Angeles Forner-Giner, & Salvador, 2021), with levels of 5.8, 2.8 and 19.6 μg·g^−1^ in SH, GS, and CH, respectively. In the low-anthocyanin budsport variety of Tarocco, and under the same assay conditions, cyanidin-like anthocyanin was 17.5 μg·g^−1^ (Zhang et al., 2025), which is lower than those of the normal blood oranges “Tarocco” and “Moro” (Morales et al., 2021). In addition, the total amount of the purple anthocyanins delphinidin-glucoside-feruloyl-xyloside and delphinidin-3-O-(coumaroyl)-glucoside-5-O-galactoside, and the blue-purple petunidin-3-O-5-O-(6-O-coumaroyl)-diglucoside was 6.4, 6.5, and 8.0 μg·g^−1^ in SH, GS, and CH, respectively, which was much higher than in “Tarocco” and “Moro”. Therefore, the redness of red pummelos may be the result of both lycopene and anthocyanins.

Recently, an individual pummelo tree with purple/red-skinned fruits was discovered in a mountainous region of Hubei Province, China (Butelli et al., 2017). Gene sequencing of *Ruby*, a key transcription factor for anthocyanin synthesis, in four pummelo varieties, including this purple-skinned pummelo, revealed that all contained at least one intact copy of the gene (Huang et al., 2019). Therefore, pummelos appear to have the potential to produce anthocyanins. However, anthocyanin pigmentation has never been reported in pummelo flesh. Previously, the red color of pummelo flesh was attributed to high levels of lycopene, and the presence of anthocyanin was not investigated further (Hasan et al., 2024; Promkaew et al., 2020; Wang et al., 2019). The results of this study indicate that the anthocyanin content of red flesh pummelos is not negligible, especially in CH, a cultivar from a local seedling selection in Lishui, Zhejiang Province, China (Yan et al., 2018). The CH contains a total of 28.5 μg·g^−1^ anthocyanin in its flesh, making it suitable as potential parental material for breeding.

In previous studies, citrus was classified into β-cryptoxanthin-, violaxanthin-, and lycopene-abundant varieties (Ikoma et al., 2016; Ma et al., 2023). In this study, six cultivars, including the red and pale-red pummelos, were classified as lycopene-abundant varieties. In contrast, the orange pummelo JJM, the orange budsport variety of Guanximiyou, had carotenoids consisting mainly of β-carotene and α-carotene (C. Jiang et al., 2019; Pan et al., 2021; C. Zhu, Lu, et al., 2020). JJM does not fit into any of the three classifications mentioned above, and a new classification, β-carotene-abundant varieties, could be defined based on this cultivar. In addition to carotenoids, JJM fruits contain more than three times the amount of flavonoids compared to the red and pink pummelos. Flavonoids can also produce a yellow color, albeit not as saturated as carotenoids, and should not be ignored. Therefore, the orange color of JJM likely results from a combination of β-carotene, α-carotene, and flavonoids.

Similar to many pummelo cultivars previously evaluated, narirutin was the most abundant flavonoid among the seven pummelo cultivars examined in this study (Lu & Wang, 2024; Multari, Licciardello, Caruso, & Martens, 2020). Narirutin is useful for the treatment of obesity, type 2 diabetes, high blood pressure, and metabolic syndromes (Multari et al., 2020). Naringin was not detected in any of the varieties, and only neohesperidin and poncirin were detected in rutinoside flavanones. These two flavonoids also belong to *O*-methylated flavonoids, and their trends in the different varieties were similar to those of several PMFs, suggesting that their methylation may be regulated by similar enzymes (Ben Hmidene, Smaoui, Abdelly, Isoda, & Shigemori, 2017; B. Peng et al., 2023). Although their content in pummelo flesh was negligible compared to that of naringin, these were the only flavonoids in this study that were significantly and positively correlated with antioxidant capacity, suggesting that *O*-methylation confers strong antioxidant activity to these flavonoids (Karn et al., 2021; Y. Liu, Qian, et al., 2022; Zhao et al., 2020).

In addition, lycopene is also a powerful antioxidant that protects proteins, lipids, and DNA from oxidation (Durairajanayagam, Agarwal, Ong, & Prashast, 2014; H. Wu, Wu, Cui, & Hu, 2023). Additionally, lycopene can act on free radicals such as hydrogen peroxide, nitrogen dioxide, and hydroxyl radicals (Durairajanayagam et al., 2014). Furthermore, the lycopene activity against free radicals is increases in the presence of other carotenoids such as β-carotene, phytoene, and phytofluene. Among all the carotenoids, lycopene had the highest scavenging ability for singlet species of oxygen free radicals (H. Wu et al., 2023). In this study, lycopene was not only the most abundant carotenoid, but also the most highly correlated with antioxidant capacity. The shade of red color of the flesh was also highly correlated with antioxidant capacity. The regulatory mechanisms affecting the red color and lycopene content in citrus fruit are not as well studied as anthocyanin content. For this reason, it would be useful to understand the mechanism of lycopene biosynthesis in citrus and the pigmented pummelo varieties described in this study could be important for further dissecting the basis of lycopene accumulation in pummelo flesh.

## 5 Conclusion

**Fig. 9:**
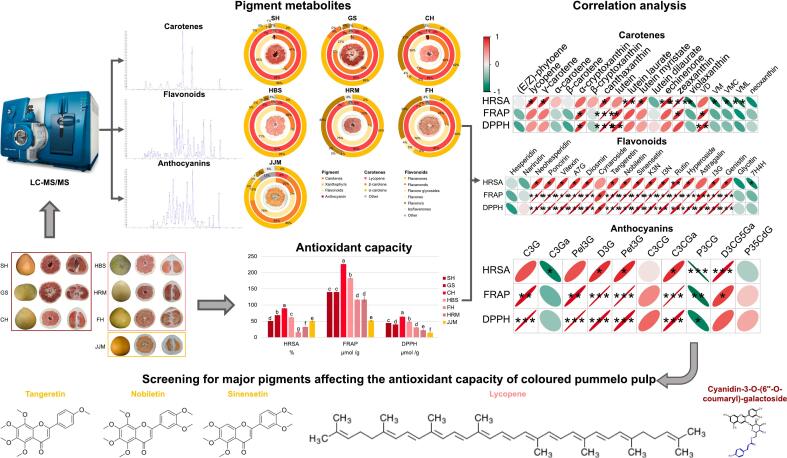
In this study, we selected seven pummelo cultivars whose pulp coloration could be classified into three color types: the red cultivars Sanhongmiyou (SH), Gusangyou (GS), and Chuhongyou (CH); the pale-red cultivars Ruby pummelo (HBS), Red flesh Guanximiyou (HRM), and Feihongyou (FH); and the orange cultivar Jinjumiyou (JJM). Determination of carotenoid, flavonoid and anthocyanin fractions in pummelo pulp by LC-MS. The narirutin and lycopene were the most abundant carotenoids and flavonoids, respectively, in the three red and three red-pale pummelo cultivars and β-carotene was the most abundant carotenoid in JJM. Cyanidin-3-O-(6”-O-coumaryl)-galactoside was the most abundant anthocyanin in three red pummelo cultivars SH, GS, and CH. Combining the antioxidant capacity of different cultivars, screening of PMFs (tangeretin, nobiletin, and sinensetin), lysopene, and cyanidin-3-O-(6”-O-coumaryl)-galactoside pigment substances affecting the antioxidant capacity of pummelo pulp. (For interpretation of the references to color in this figure legend, the reader is referred to the web version of this article.)

In this study, we examined the carotenoid, flavonoid, and anthocyanin content in the flesh of seven colored pummelo cultivars, SH, GS, CH, HBS, JJM, FH, and HRM, using LC-MS/MS. We found that narirutin and lycopene were the most abundant flavonoids and carotenoids, respectively, in the three red and three red-pale pummelo cultivars, and β-carotene was the most abundant carotenoid in JJM. Furthermore, cyanidin-3-O-(6”-O-coumaryl)-galactoside was the most abundant anthocyanin in three red pummelo cultivars SH, GS, and CH. Given our findings, the shade of red of pummelo flesh is likely determined by the combination of lycopene and anthocyanins, while the orange color of the JJM is primarily due to a combination of β-carotene, α-carotene, and flavonoids. Additionally, we examined the antioxidant capacity of different cultivars, identifying several key metabolites that potentially affect the antioxidant capacity in pummelo flesh, such as PMFs (tangeretin, nobiletin, and sinensetin), lycopene, and cyanidin-3-O-(6”-O-coumaryl)-galactoside. This study not only highlights potential metabolites affecting color formation and antioxidant capacity in colored pummelo flesh but also provides valuable insights for the development of citrus resources (Fig. 9).

## CRediT authorship contribution statement

**Peian Zhang:** Writing – review & editing, Funding acquisition, Writing – original draft, Conceptualization, Visualization, Data curation, Formal analysis. **Quan Zhao:** Data curation, Funding acquisition. **Yang Song:** Data curation. **Huanchun Jin:** Investigation, Data curation. **Danelle Seymour:** Writing – review & editing. **Yingyao Liu:** Data curation. **Jun Chen:** Data curation. **Dan Hu:** Investigation. **Dongfeng Liu:** Funding acquisition, Investigation, Writing – review & editing, Conceptualization.

## Declaration of competing interest

The authors declare that they have no known competing financial interests or personal relationships that could have appeared to influence the work reported in this paper.

## Data Availability

The data that has been used is confidential.

## Appendix A Supplementary data

Supplementary material 1 Supplementary Figures Supplementary material 1

Supplementary material 2 Supplmentary Tables Supplementary material 2
