# Supplementary material for: Evaluation of pigment composition and antioxidant properties in the flesh of seven colored pummelo cultivars
Source: Food Chem X. 2025 Jun 26;29:102705. doi: 10.1016/j.fochx.2025.102705 (PMC12270813; doi:10.1016/j.fochx.2025.102705)
Supplement: Supplementary material 1 — Supplementary Figures [file mmc1.docx]

Fig. S1 PCA plots for flavonoid (A) and carotenoid (B) metabolomics of ripe pulp of seven pummelo cultivars. PC1 (principal component 1) explained 28.55% and 31.63% of the total variance, respectively. While PC2 (principal component 2) explained 21.17% and 26.23% of the total variance, respectively.


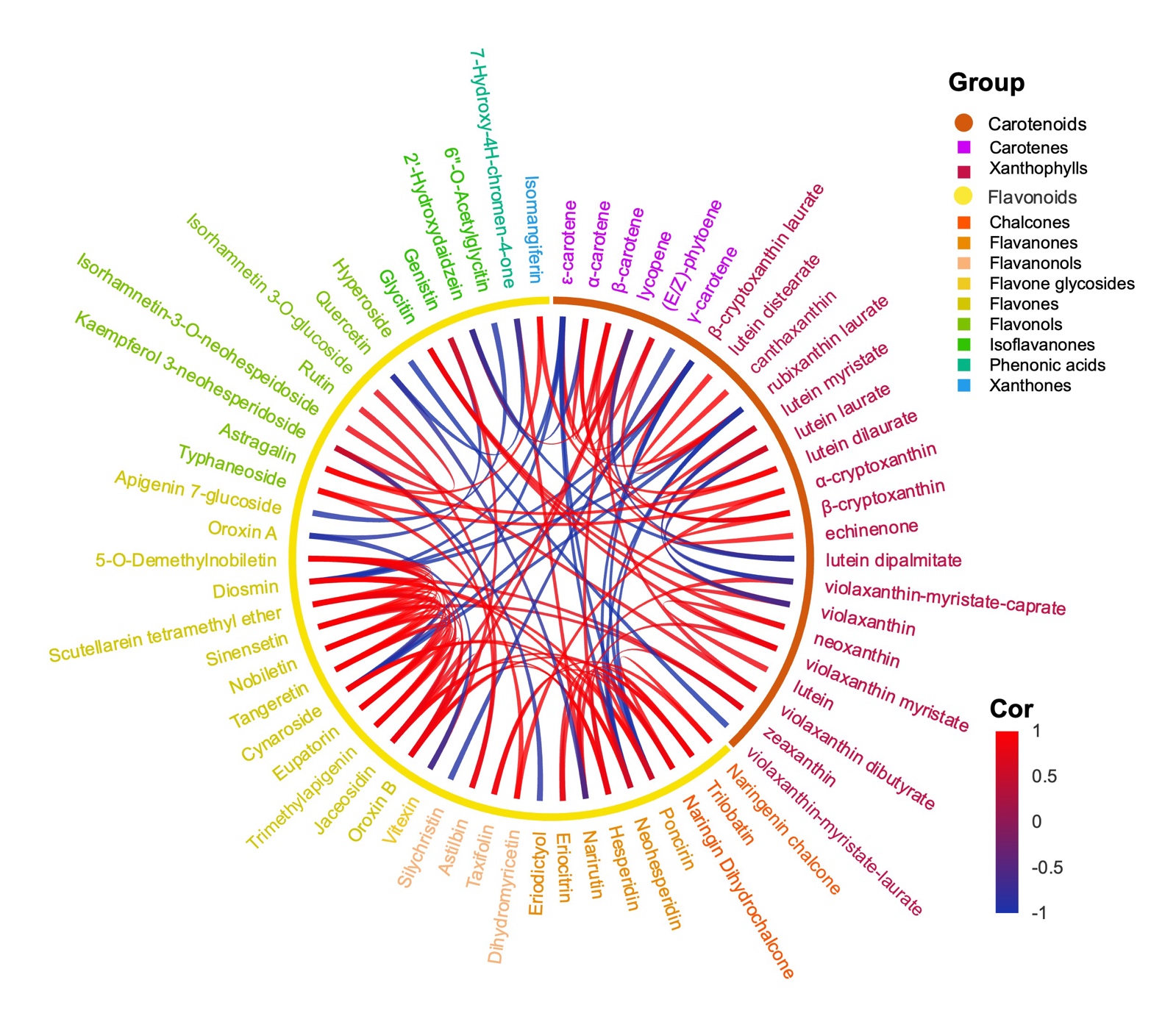


Fig. S2 The Pearson correlation of key flavonoid and anthocyanin metabolites of seven pummelo cultivars. The metabolites linked in chord plot indicate significant correlations (R^2^>0.8, *p*<0.05). The color scale in the lower right corner shows Pearson correlation from −1 (red) to 1 (blue).

Fig S3 OPLS-DA plots (A, B), PCA plots (C), and circos relationship plots (D) of three red pummelo cultivars. A and B are the OPLS-DA score plots and S-plot of anthocyanin in the pulp of the three cultivars, respectively. C are PCA plots for anthocyanin metabolomics of three cultivars. D are circos relationship plots for different class anthocyanin metabolomics of three cultivars.

Fig. S4 The Pearson correlation analysis between antioxidant capacities (HRSA, FRAP and DPPH) and color indicators (L, a, b, H, and CCI). The colour scale in the right corner shows Pearson correlation from −1 (green) to 1 (red). ‘*’, ‘**’, and ‘***’ indicate *p* values <0.05, 0.01, and 0.001, respectively. Abbreviations: HRSA, Hydroxy free radical scavenging activity; FRAP, Ferric reducing antioxidant power; DPPH, 2,2′-diphenyl-1-picrylhydrazyl free radical scavenging capacity; CCI, citrus color index; H, hue angle.
